# Experience and Acceptability of a Reduced-Energy Whole-Diet Intervention in Women with Gestational Diabetes: a Qualitative Study of the Dietary Intervention in Gestational Diabetes Trial

**DOI:** 10.1016/j.cdnut.2025.107633

**Published:** 2026-01-05

**Authors:** Laura C Kusinski, Sarah Dib, Suzanne Smith, Danielle L Jones, Amy L Ahern, Claire L Meek

**Affiliations:** 1 Leicester Diabetes Centre and Leicester National Institute for Health and Care Research (NIHR) Biomedical Research Centre, University of Leicester, Leicester General Hospital, Leicester, United Kingdom; 2 Institute of Metabolic Science – Medical Research Laboratories, University of Cambridge, Cambridge, United Kingdom; 3 Medical Research Council (MRC) Epidemiology Unit, University of Cambridge, Cambridge, United Kingdom; 4 Cambridge Universities National Health Service Foundation Trust, Cambridge, United Kingdom; 5 University Hospitals of Leicester National Health Service Trust, Leicester General Hospital, Leicester, United Kingdom

**Keywords:** pregnancy, gestational diabetes, qualitative research, food intervention, energy restriction

## Abstract

**Background:**

Gestational diabetes mellitus (GDM) is rising in prevalence in many countries worldwide. Although dietary modifications are the first-line treatment for GDM, it is unclear which specific dietary approaches are most effective to improve maternal and offspring outcomes while reducing further maternal weight gain. We recently demonstrated benefits of an energy-restricted diet with modest weight loss in women with GDM.

**Objectives:**

We aimed to investigate the acceptability of an energy-restricted whole-diet intervention in women with GDM with a BMI ≥25 kg/m^2^, in a free-living environment, and to examine potential barriers and enablers of implementation of such a diet into standard clinical care.

**Methods:**

This qualitative research study used semistructured interviews with 20 participants in the Dietary Intervention in Gestational Diabetes trial, in which women with GDM were randomized to a whole-diet intervention containing 1200 or 2000 kcal/d. We used reflexive thematic analysis to explore participants’ experiences of the study diet and GDM.

**Results:**

We identified 6 key themes in participants’ experience: emotional and cognitive burden of managing GDM, convenience of meal delivery, support and reassurance, hunger and appetite differences, health impacts for intervention engagement, and research participation for awareness and impact. Facilitators of study participation included support, motivation, convenience, and not wanting to be medicated. Barriers to participation were the social aspects of eating food, inconvenience, and uncertainty about the validity of dietary interventions. It was identified that participants frequently overlooked their physical health and weight management during pregnancy, despite how crucial these factors are for both maternal and infant well-being.

**Conclusions:**

Pregnant women with GDM found an energy-restricted diet to be acceptable and beneficial to emotional and physical health, especially when support is provided by healthcare professionals. The convenience of meal delivery and the motivations to avoid medication use in pregnancy facilitated engagement with the intervention.

This trial was registered at ISRCTN registry as ISRCTN 65152174 (https://www.isrctn.com/ISRCTN65152174).

## Introduction

Gestational diabetes mellitus (GDM) affects ∼14% of pregnancies globally, and this prevalence is increasing with rising rates of type 2 diabetes and obesity [1]. Maternal hyperglycemia has been associated with adverse pregnancy outcomes and higher long-term cardiometabolic risk in women and children exposed to GDM [[2], [3], [4], [5], [6]].

Medical nutrition therapy is the first-line treatment for GDM with the primary goal of preventing maternal hyperglycemia. Dietary approaches for GDM are associated with some improvements in perinatal outcomes, but the evidence is inconclusive on whether one approach is better than the other, and the need for higher-quality studies remains [7].

Restricting gestational weight gain has the potential to reduce adverse outcomes associated with GDM or maternal obesity. The Dietary Intervention in Gestational Diabetes (DiGest) trial aimed to identify whether energy restriction using a whole-diet intervention, in women with GDM and a prepregnancy BMI ≥25 kg/m^2^, improved pregnancy outcomes. Participants were recruited at ∼28 to 32 wk’ gestation and randomized to receive a standard-energy dietbox (2000 kcal/d) or a reduced-energy (RE) dietbox (1200 kcal/d). The main findings showed that the RE diet in late pregnancy decreased the requirement for long-acting insulin. Further analysis showed those who lost weight, irrespective of dietary intervention arm, during this period had the most marked clinical benefit with improvements in maternal glycaemia and a reduction in the large-for-gestational age rate [8].

We previously demonstrated that healthcare professionals caring for women with GDM found the DiGest dietboxes were convenient and could be incorporated easily into their daily life (work/home settings) [9]. The aim of the current study was to assess the acceptability of a whole-diet intervention in women with GDM in a free-living environment and identify potential barriers or enablers to implementation of such a diet in standard clinical care.

## Methods

### Design and setting

This qualitative study of participants’ experiences was an optional ancillary study offered to participants of the DiGest trial at the end of the trial period. The DiGest trial was conducted according to guidelines from the Declaration of Helsinki, and ethical approval was obtained by the West Midlands Research Ethics Committee (reference 18/WM/0191) and the National Health Service (NHS) Health Research Authority (IRAS 242924). The trial was listed at the ISRCTN registry (65152174).

### Recruitment and participant selection

Participants were eligible for the DiGest trial if they were aged ≥18 y old and diagnosed with GDM before 30 + 6 wk of gestation, had a singleton pregnancy, a prepregnancy BMI ≥25 kg/m^2^, no major preexisting comorbidities, and no other serious pregnancy complications.

Participants were approached to take part in this optional qualitative assessment by e-mail, through a participant information leaflet detailing the purpose behind the study. Additional consent was obtained from the interested participants for their involvement in the interview and audio recording. Online interviews were arranged at the earliest convenience of the participant. One participant declined the audio recording for the interview but allowed the interviewer to make detailed notes during the interview. On the basis of previous experience of similar qualitative studies [9,10], we aimed to interview between 15 and 20 participants with roughly even numbers across the 2 intervention arms to ensure rich data quality was achieved from participants in both groups.

### The DiGest trial intervention

Participants had baseline measurements taken between 28 and 30 wk gestation and then were randomized to a standard-energy diet (2000 kcal/d) or RE diet (1200 kcal/d), provided free of charge, and were blinded, along with their health care professionals, to their allocation for the duration of the trial. Participants remained on their allocated trial arm until delivery. Participants had access to a study smartphone for the duration of the study if it was required. Study visits were conducted at 32 and 36 wk of pregnancy and 12 wk postpartum. Some of the trial participants were on the study during the COVID-19 pandemic; as such, some of the research visits had to be scaled back to fall in line with social distancing guidelines. Interviews were conducted after this final study visit had already taken place. Trial arm assignment was not known to the participants during the interview. Participant flow chart can be seen in Supplementary Figure 1.

### Data generation

Semistructured qualitative interviews were performed from July 2020 until August 2024. This approach enables the participants to provide more in-depth responses and the freedom to detail specific aspects which they thought relevant to their experience [11]. The interviews were conducted by a single postdoctoral research associate (LCK) who had previous training in qualitative research. The interview schedule was developed by the study team, based on our previous qualitative study done in health care professionals [9], with additional questions added to the schedule so that all participants’ experiences would be covered. The full interview schedule can be found in the supplementary material (Supplementary Figure 2). Interviews were conducted online, audio-recorded, and transcribed with the permission of the participant. Interviews ranged from 23 to 60 min, with an average of 40 min.

### Data analysis

For this dataset, we used reflexive thematic analysis to examine patterns across transcripts. This process was deemed appropriate as it allows for participants’ thoughts and experiences to be thoroughly evolved from a broad framework into themes which can be interpreted. This was conducted according to the 6 phases of thematic analysis as outlined by Braun and Clarke 2021. These phases include familiarization, coding, generation of initial themes, developing and reviewing themes, refinement and defining themes, and finally producing the report [12]. This was done using an iterative approach by 2 researchers (LCK and SD).

Each interview transcript was read several times by both researchers to allow them to familiarize themselves with the content, and notes were made of key aspects. After this, the 2 researchers developed independent codes for the dataset. These codes were then discussed, and a collaboration of these codes was generated to determine a master coding file. Interview transcripts were then divided and coded independently (LCK = 11; SD = 12; 6 transcripts were double-coded). After the detailed coding of the transcripts, researchers developed themes of the dataset through detailed discussion and interpretation of related codes. This collaborative coding approach supports each researcher’s reflexive engagement with the data. The developed themes are the overarching categories that detail the participants’ experience of the intervention, and the results are detailed in the results and discussion section.

## Results

Twenty DiGest participants took part in the qualitative interview, including 9 from the standard-energy and 11 from the RE intervention arm. The baseline characteristics of the main study and this subset of participants can be seen in Table 1. The time of the interview varied for each participant, with a mean of 389 d after birth (range: 90–1176 d).

**TABLE 1:** Baseline characteristics of participants.

	All participants (*n* = 425)	Standard-energy (*n* = 9) (2000 kcal/d) Mean (SD) or *n* (%)	Reduced-energy (*n* = 11) (1200 kcal/d) Mean (SD) or *n* (%)
Age (y)	33.03 (5.04)	36.7 (4.2)	33.9 (5.2)
Baseline weight (kg)	95.4 (20.1)	97.5 (19.5)	84.0 (16.8)
Baseline BMI	35.7 (6.4)	36.0 (6.5)	30.8 (5.1)
Primiparous	136/425	4/9	5/11
Weight change (29–36 wk) No weight loss	235/389 (60.4%)	5/9 (56.0%)	4/11 (36.0%)
Weight loss	154/389 (39.6%)	4/9 (44.0%)	7/11 (64.0%)
Ethnicity			
White	289/425 (68.1%)	6/9 (67.0%)	7/11 (64.0%)
White (other)	43/425 (10.1%)	3/9 (33.0%)	0/11 (0%)
Black/Black African	17/425 (4%)	0/9 (0%)	2/11 (18.0%)
Asian	73/425 (17.2%)	0/9 (0%)	2/11 (18.0%)
Highest education status			
Postgraduate qualification	67/425 (15.8%)	4/9 (44%)	4/11 (36%)
Undergraduate degree	134/425 (31.5%)	1/9 (11%)	4/11 (36%)
A-level or equivalent	127/425 (29.9%)	4/9 (44%)	3/11 (27%)
Number of weeks enrolled in the intervention	10.5 (1.8)	10.9 (1.3)	11.1 (1.0)

We developed 6 themes through detailed thematic analysis of the transcripts. These are described below, and quotes supporting each theme can be found in Table 2.

**TABLE 2:** Quotations from DiGest trial participants for each theme. Each quote is followed by the ID of the participant and the intervention arm they were assigned.

Theme	Quotations from participants *n* = 20
1. Emotional and cognitive burden of managing GDM	A. “I think it’s an extremely stressful thing to go through and it’s sort of like having to think about doing your own food and stuff and what’s, you know, all of that is just like (sighs), like way too much to expect of women, you know, and it just causes them a lot of stress.” 3102, RE B. “First of all, I was very shocked cause I did not expect to have the gestational diabetes. Bearing in mind that there’s no history of any diabetes in my family and I had no signs.” 9023, RE C. “I still found it really overwhelming to try and work out what I could and couldn't eat, and it just felt like another thing that I was responsible for.” 3194, RE D. “When you’re given the diagnosis of diabetes, it’s very daunting at the beginning and having to think about what you can and can’t eat.” 7013, RE E. “let’s try and be sensible, even though I really want an ice cream. I won't have it.” 3121, RE F. “Probably having fewer carbohydrates, more protein and more vegetables than I was before. Whereas I think in the past I probably would have had quite a lot more carbs.” 3194, RE G. “I don't think there is a lot of support for gestational diabetes out there. They kind of put you on a monitor and they make you check your blood sugar concentrations and insulin if necessary. But the cause of a lot of people’s gestational diabetes is food, and if you don't know how to change that and you're not able to change that, then actually that’s no help the mothers anyway.” 3121, RE H. “…The information trying to actually work out what was truthful and not to help my body and the baby was really hard to, I don’t know, digest probably…If I’m honest, I wasn’t given much of any useful dietary advice at all…….It was a trial and error of what foods are working and OK and what aren’t.” 5003, SE
2. Convenience of meal delivery during GDM	A. “You didn’t have to be doing any thinking; you could literally go anywhere, and you just took it with you.” 7013, RE B. “I didn’t miss cooking at all, it was very handy, it came at the right time, because I had just moved house, I was working, I didn’t need to worry about what I was going to eat.” 2032, RE C. “Bearing in mind with the diabetes…sometimes I was sickly, sometimes I was tired, so if I’m told to go to the kitchen and prepare a meal, I have to count all the calories, I have to do all these, I mean, I’ll just be thinking of, I’m hungry, I just need something to eat. So having them (the meals) ready, it was much more beneficial.” 9023, RE Social challenges of dietary adherence D. “At weekends when we’d go round family members and they’d be having like a takeaway and things like that, that was kind of hard, because it was like I can’t have what they’re having” 3015, RE E. “I think where it was sessions where we were meeting people like friends and family for the one meal that we would have out, I would come off the diet. But then sometimes because I was trying to stick to the DiGest meals. I would sometimes say, well, we’ll go out for maybe like a coffee and I'd have like a cup of tea or something like that, and I wouldn't have a cake. So I’d go out for times where you wouldn’t have to have a meal.” 3121, RE F. “On my birthday and one other night I did go to a restaurant and not follow a diet. But in general, if I went around to somebody’s house for dinner, I’d take my meal with me and get them to cook it for me. So yeah, it worked.” 7013, RE
3. The value of additional support and reassurance	A. “It was great to be able to be like I can just have this food. This is what I’m eating and I don't have to worry or think like because somebody has worked out that this is good and I can have it.” 3194, RE B. “Not having to think about preparing and doing food, particularly when it was it had to change and it. I had to have specific food. So the fact that it was all done for me was probably the main reason that I did it.” 9008, RE Support from the research team C. “That little cheerleader behind the scenes somewhere just to go look, we know we can, we’re looking at your numbers and you’re doing it. Keep going. You’re doing it just sort of, especially when you’ve got your baby in mind, gives you that push to keep going.” 5003, SE D. “Getting that support, of support, that made it easy for me to just crack on with the whole program.” 2032, RE E. “That support there to actively help me, so I knew that I was going to stick to it.” 2032, RE F. “I liked the whole monitoring system, I had this device on my arm, I knew there was someone or something watching me at the other end, so I was careful with what I was putting in.” 2032, RE G. “It probably would have been helpful because those first few months are always a little bit tricky for sleep, so having something pre laid out you don't have to worry about the shopping would have probably been useful just from a practicality point of view to.” 7015, SE The importance of social networks H. “I think loads of people were really interested as well, and I think that made it quite nice because like telling them what I was having for lunch, I think people found it really interesting that the study was happening.” 3011, SE I. “People were interested in what I was having, so it got me talking about the study and what I was doing so that was quite nice as well.” 9009, RE J. “I think my friends kind of got the hang of it and my family got the hang of it after a while. Particularly my parents are really impressed with how much I stuck to it when I went there ‘cause, like I said, my family are feeders and they are like, sure you don’t want anything? And I was like, no, this is, you know, I have to have to do this… Everyone was really supportive. They were amazed I could stick to it. My family were really impressed that I did it.” 9009, RE K. “I don’t think I necessarily missed cooking, but I missed sort of like the culture of sitting at the table with my husband and eating the same meal.” 3194, RE L. “If I was cooking dinner, I’d try and cook something similar to what I was having for them so that it was like we were having the same meal, but mine was obviously from a from a frozen meal.” 7015, SE M. “It was a bit frustrating for me because it felt gave a lot of people license to talk about my body. People kept saying you look amazing, but and I was like, oh, do you know, actually I don’t find it that comfortable to discuss weight loss like that, you know?” 3194, RE
4. Individual differences in hunger and appetite	A. “I wasn’t hungry at all. The portions were quite good.” 3011, SE B. “I was really hungry all the time and sometimes it was quite difficult.” 3102, RE C. “The varieties were not a lot bearing in mind I had a lot of gastric hyperacidity... and I had to stick to the same type of foods over and over again. So that was the only challenge that I encountered.” 9023, RE D. “Initially, our family hesitated, in our culture, once you’re pregnant, you need to be eating something extremely healthy for nutrition and eating frozen food is definitely not an option that will be considered at all.” 3171, RE E. “I was slightly concerned…was I going to spend the next 6 months starving or what was that going to look like without any real understanding of the types of foods included.” 5003, SE
5. Health impacts for mother and baby as a motivator for intervention engagement	A. “I knew, to be fair, I wasn’t in my healthiest best so restricting weight gain was definitely not a concern. Had it been dramatic…I would have hoped that I had enough people seeing me that they’d have gone right, OK, this is now becoming a concern, we need to watch it.” 5003, SE B. “I felt very like looked after in a sense. So I felt as though all that extra monitoring meant as well that somebody would spot a problem and intervene if needed, so I wasn't concerned that I was losing too much weight or anything like that.” 3194, RE C. “I know that even though my weight isn’t gaining, doesn’t mean it’s my baby is not growing.” 3171, RE D. “There weren’t any bad sides or any negatives that I was aware of because it wasn’t like putting chemicals into my body. It was just food. So which I’m taking part in it anyway, so I think I knew the risk would be very, very low to the baby.” 5003,RE E. “I think it raised my awareness of, you know, like what sugars are in what kind of thing. It’s made me think more about what I eat. I don’t know what effect it had on him, really. He was a normal birth weight.” 8026, SE F. “I have a sweet tooth and that’s not helpful when you the likelihood of getting, you know, diabetes in the future so.” 9009, RE G. “It (taking part in the study) didn’t necessarily change my mindset. I think I was focused on it being a pregnancy thing and then after I came back to normal.” 9006, RE H. “As soon as you have your baby like you’re off the study and then your world just explodes and it’s chaos and you’re craving sugar because you’re tired and or your brain is anyway it’s like feed me and so I think I sort of you almost like just drop off a cliff and or that’s how it felt maybe and then it was still COVID. I ended up being quite sedentary. And so I think it sort of then snowboard and spiraled. And rather than being able to continue on a good and healthy path, I sort of just sort of just stagnated.” 5003, RE I. “I have been trying to like have smaller portions and you know, don't do second helpings and stuff like that.” 9009, RE J. “We eat quite fresh food now. So, we’re eating a lot of fresh fruit, we eat a lot of like chicken and beef and things like that. So, more protein, less carb in the house, which is really good, and my partner’s enjoying having my meals. My portion sizes weren’t great beforehand, but now they’re a lot smaller which he’s finding it’s helping him to continue to sort of losing weight as well as me maintaining my normal weight.” 3015, RE K. “Knowing what snacks I can have in the day you made it better and that’s something I'm going to try and do.” 9009, RE L. “I was somebody before this study who didn't regularly breakfast………..but actually because of the study, I was eating breakfast regularly…….. and I think that that made a big change and that was something that I carried on so that when my son was here and I was off to study, I've still carried on having breakfast.” 3121, RE M. “I never used to eat breakfast before, but now I’m intentionally eating breakfast…..I’ve continued what I was doing while I was pregnant.” 2032, RE N. “We walked 10k yesterday, we walked 10k last week.” 9026, SE Avoiding medication O. “My husband was super-supportive in that he, you know, he also completely agrees that if it wasn’t for being on that I might have had to go on metformin, like he totally agrees, he thinks it’s really, really fortunate I was on the study to help me manage it, to stay diet controlled.” 3102, RE P. “The motivator for sticking to it, was the fact I didn’t want to go on insulin.” 9026, SE Q. “I was like really grateful to be on the study because I remained diet controlled my whole pregnancy and yeah, I often think, like I don’t think, I think it would have been much harder for me to remain diet controlled under my own like devices.” 3201, RE R. “I knew I was going to end up having metformin or insulin, I saw that coming, I saw that coming. Somehow even though I joined the diet late, the study late, I still managed to avoid that, that route. So I’m very happy about the fact that I joined the diet. So I wouldn’t have been able to do it myself.” 2032, RE
6. Research participation for awareness and impact	Increased support and motivation A. “Just being part of anything that brings a bit more awareness and a bit more understanding to what people go through with gestational diabetes and how to support mothers ‘cause? I don't think there is a lot of support for gestational diabetes out there.” 3121, RE B. “That sounds stressful, but here you could have this and that sort of should help manage it without you having to be. Like without you having to take the responsibility. And I think that that for me was like just exactly what I needed. So yeah, it took a lot of, took a lot of anxiety away for me.” 3194, RE C. “It really helped me also mentally, to turn something that I had a lot of trouble to deal with to start with, having gestational diabetes on, in a positive experience. So, because when I was thinking, oh, it sucks to be, to have diabetes, I thought, well, at least something positive will come out of it, I will be part of the study. So, yeah, it really improved my morale toward the end.” 9031, SE Involved in research and impact D. “In the sort of grand scheme of things, I thought if I could help people out in the future who have gestational diabetes and I could play my part in helping future understanding…I notice the understanding of it at the minute is very limited, the research and the knowledge on it is quite, even the NHS, is quite dated from what I can gather, so if I can help bring that along and help women in the future who get this, then I’ll definitely want to play my part in that.” 7013, RE E. “I was strict on myself because I wanted to do it properly…I think the fact you're doing a study and it’s for research and it’s for helping other people in the future, that was my main incentive to be really, really good and follow it.” 9009, RE

Abbreviations: DiGest, Dietary Intervention in Gestational Diabetes; GDM, gestational diabetes mellitus; NHS, National Health Service; RE, reduced-energy; SE, standard-energy.

### Theme 1: the emotional and cognitive burden of managing GDM

Participants reported that being diagnosed with GDM was a stressful experience (Table 2, Quote 1A). Participants often described receiving a GDM diagnosis as an unexpected event that they had not anticipated (Table 2, Quote 1B), despite having risk factors for the condition.

In addition to feeling stressed and overwhelmed about the diagnosis, many participants described uncertainty about what the diagnosis meant in terms of food intake and how to manage the condition. This was perceived as an additional burden that they had to take responsibility for, and which caused additional stress (Table 2, Quote 1C and D).

Despite being a stressful period in pregnancy, some participants were able to take control of self-management and became very aware of the food they were consuming (Table 2, Quote 1E and F). Participants reported making more deliberate decisions not to eat certain things to try and achieve optimal glucose concentrations. This was particularly relevant with the dietary intervention, as this was able to lessen the burden of food choices for the participants, as the food choices had already been made for them by the food provision. Improved awareness of food consumption was an important driver of intervention adherence and resulted in a willingness to follow the intervention.

Participants recalled their experiences of diagnosis and would reflect on this period as one where support is limited. They described feeling overwhelmed during this time and often not supported by the health care professional team in a way that they would like, and this had an impact on their emotional/mental health (Table 2, Quote 1G).

The participants referred to the advice that they were given to try and control their blood sugars as often conflicting and not clear for them to understand. This limited information meant that they were relying on online advice or managing the condition independently by trying to establish what foods worked best for their own personal blood sugar concentrations (Table 2, Quote 1H). As a result, participants approached the intervention with a heightened sense of uncertainty, but viewed the provision of the food as an opportunity to obtain more reliable, structured, and tailored dietary guidance that aided their acceptability of being on the intervention.

### Theme 2: the convenience of meal delivery during GDM

In light of their GDM diagnosis and the burden that ensued with this, the participants highlighted that the convenience of the pre-prepared meals was a major benefit of being in the study that was greatly valued (Table 2, Quote 2A). Participants overwhelmingly reported that having preprepared meals was easy to incorporate into daily life, reduced mental load and effort, and provided convenience that allowed them to continue with the diet during pregnancy. They noted that the pre-packaged meals required little to no preparation, with comments like “all I needed to do was just put them in the microwave” (2032, RE), illustrating the minimal effort needed to prepare each meal. This also meant that it was suitable and adaptable to their daily routines. The convenience of the preprepared meals was highlighted in both intervention arms as a huge benefit of being in the study and as such played an important role in intervention adherence.

Many appreciated the relief the dietboxes provided by reducing the mental effort needed to plan the meals, count calories, or think about what would be suitable for diabetes (Table 2, Quote 2B and 3C). This was especially true for participants with additional stressors and responsibilities other than managing their GDM, such as busy work schedules, childcare or moving houses. Comments such as “it was one less thing for me to think about” (9006, RE) and “it came at the right time” (2032, RE) reflect how these dietboxes alleviated stress and the mental and physical load during demanding periods.

The incentives that the study provided were also motivating factors for the participants. For example, participants noted that the ready-to-eat meals “definitely saved a lot of time and money” (7013, RE). Some participants also appreciated the extra surveillance that was provided as part of the study procedures, noting that “if something was wrong, it would have been detected very easily through the study... so I think that was also one of the benefits that I enjoyed.” (9023, RE).

Although the convenience of the dietboxes was evident in this population, it is important to address the social challenges of dietary adherence that were also felt by many of the participants. Given that the intervention lasted for ≤10 wk during the last phase of pregnancy, the participants had to navigate their diets over holidays and weekends, which they found more challenging than workdays (Table 2, Quote 2D). Participants mentioned that they tried to overcome this barrier or cope by taking their meals with them when they went out, eating food that is similar to the provided DiGest meal, or avoiding social gatherings that involved food altogether. However, on a couple of social occasions, some participants diverged from the DiGest diet (Table 2, Quote 2E and F).

### Theme 3: the value of additional support and reassurance

The DiGest trial provided women with gestational diabetes appropriate meals, which in turn empowered them to feel more in control (Table 2, Quote 3A). “Being in control” was a key element of this intervention. The dietboxes provided by the study meant participants were able to see what the recommended food types and portion sizes were, with minimal planning required on their part (Table 2, Quote 3B), reducing the cognitive burden of food selection and meal planning.

#### Support from the research team

The additional support provided to the participants from the research team was highlighted as a positive factor by the women interviewed (Table 2, Quote 3C and D). Although visits were aligned with antenatal appointments, the extra time spent with participants during these visits and the additional support through them being able to have regular contact with the research team (e-mails, texts, and phone calls) enabled them to feel cared for and supported in their pregnancy. The participants’ ability to stick with the diet was closely tied to the extra reassurance and support by the research team (Table 2, Quote 3E). This encouragement was a major motivator, underscoring that adherence depended not only on the diet itself but also on the support that was provided.

Participants greatly appreciated the extra monitoring that was provided as part of the trial (Table 2, Quote 3F). In particular, the additional surveillance of their blood sugar glucose through continuous glucose monitors provided them with reassurance. Additional face-to-face study visits with the research team gave participants the platform to raise any queries and provided them with emotional support during the pregnancy. Taken together, this extra contact, along with the extra monitoring of participants and their babies, provided reassurance, which helped to alleviate anxiety.

However, participants often commented on there being a lack of support by health care professionals post delivery, and included in this was the fact that the food intervention ceased (Table 2, Quote 3G). Some participants stressed that this period was often difficult with a new baby and would have preferred for the dietboxes to continue during this time to help them with their diet while navigating parenthood.

#### The importance of social networks

There was also an overwhelming sense of support received by the participants from their families, friends, and colleagues during the study. Participants mentioned that there was often curiosity about what the study involved, but also encouragement and recognition of the value of participating in it (Table 2, Quote 3H). This support made being in the study, and particularly on a special diet, less isolating and more motivating for the participants as it allowed them the opportunity to share their experiences (Table 2, Quote 3I). The support also meant that the challenges of the change in family and social eating habits (e.g., eating different food from the rest of the family, eating out with friends or at work) were easier to navigate (Table 2, Quote 3J).

Mealtimes with their partners and/or children were another aspect that they had to navigate as they were having different food from the rest of the family, which they did not need to prepare (Table 2, Quote 3K). Some participants mentioned taking steps to overcome this, such as preparing meals that were similar to the DiGest meals as a way for the whole family to eat similar food together (Table 2, Quote 3L).

It is important to note that some participants mentioned negative aspects related to social support. For example, receiving unwanted, albeit “positive,” comments about their weight loss (Table 2, Quote 3M). This falls into the societal norms of feeling entitled to comment on others’ weight loss, praising it without understanding the context, which may be especially insensitive during pregnancy, when women may feel vulnerable.

### Theme 4: individual differences in hunger and appetite

A commonly reported challenge for the participants was the feeling of hunger. This overall ranged from participants not feeling hungry at all, to others feeling hungry throughout (Table 2, Quote 4A and B) and was independent of the intervention arm. However, overwhelmingly, those who felt hungry were able to cope by consuming snacks provided by the dietboxes, which were permitted on the diet, such as vegetables (optional additional vegetable/salad boxes were provided for participants), nuts, or cheese, and by keeping snacks for particular times in the day when hunger would more likely be a problem. This is a strategy that was adopted by many of the participants and, as such, made the hunger aspect of the dietary intervention more acceptable.

Although the general impression was that the ready-to-eat meals were convenient and easy to implement into day-to-day life, there were some instances that presented as obstacles for the participants. For example, some participants noted that “there weren’t many choices for food on the go” (9008, RE), and most meals required a microwave, which meant that they had to ensure that a microwave was available if they ate outside their homes. There was also a sense of boredom with the repetitiveness of the food options, especially for those with dietary restrictions or certain food preferences (Table 2, Quote 4C).

Some participants also expressed initial hesitancy about the diet. For instance, in certain cultures, fresh and nutritionally dense foods are seen as essential during pregnancy, and thus frozen ready-made meals were not initially considered acceptable (Table 2, Quote 4D). For others, the hesitancy stemmed from uncertainty about what consuming the RE or standard-energy diets would look like. However, these initial concerns were alleviated after receiving the food packages when they were able to see what the meals included (Table 2, Quote 4E). Despite these challenges, many participants expressed that the downsides felt like a “necessary payoff to have all of the benefits.” (3194, RE).

### Theme 5: health impacts for mother and baby as a motivator for intervention engagement

Another theme that we have identified is the participants’ perceptions of their physical health, including their weight management, and how they viewed their role in modifying pregnancy and infant outcomes, but also their physical health status postpartum.

Many participants acknowledged that they started the pregnancy or the intervention (in late pregnancy) at a higher weight. For them, this meant that they had “room to lose weight” and were not concerned about the weight loss or reduced weight gain that they may have experienced due to being in the study (Table 2, Quote 5A).

The majority of participants were unconcerned about weight loss (or reduced weight gain) during the study and felt reassured by being part of the trial. This reassurance seemed to stem from the surveillance that they were receiving and the knowledge that their baby was growing (Table 2, Quote 5B and C).

On the other hand, a few participants mentioned some negative aspects of the restricted weight gain or the weight loss that they have experienced. These included feeling hungry and receiving unwanted comments on their weight loss and appearance both previously discussed. Despite the reassurance that only a few reported experiencing a negative experience, it appeared that most participants did not consider that restricting gestational weight gain would yield a positive impact on pregnancy and birth outcomes.

Similarly, it appeared that some participants did not really consider the impact being on the diet had on their baby. They took the view that the intervention was to try and help them maintain good blood sugar readings and considered that because it was only a food intervention that this could not be harmful to the baby (Table 2, Quote 5D). However, when asked about whether they thought being in the study had an impact on their baby, most participants were not sure and often would comment that their baby was a healthy size or weight (Table 2, Quote 5E).

Although having GDM is a risk factor for type 2 diabetes, it was clear during the interview process that only some of the women had awareness that diet or weight management during pregnancy was relevant to their longer-term health (Table 2, Quote 5F). In addition, none of the women noted that their postpartum diet/weight management could impact the risk of progression to type 2 diabetes. Overall, there was a sense of lack of need to be diet conscious postpartum because the baby had been delivered (Table 2, Quote 5G).

Some participants discussed that even if they had adhered to the diet during the study, they returned to their original food habits after delivery. Some of the reasons included insufficient postpartum support, change in circumstances due to having a baby, the COVID-19 pandemic, increased hunger postpartum, or perceived need to change their intake due to breastfeeding (Table 2, Quote 5H). The fact that the meal provision was stopped at a critical time of chaos and overwhelm that comes with having a new baby, where the cognitive mental load is perhaps at capacity, was highlighted as a negative of the study. It is possible that some participants had solely used the meal provision during their pregnancy for ease and to reduce mental load, and not attempt to adapt to new eating habits.

Nevertheless, many reported a change in lifestyle postpartum due to being in the study. This has ranged from making healthier food choices during meal times and focusing on portion control (Table 2, Quote 5I and J), making a conscious effort to eat regularly at each meal time and not to skip meals (Table 2, Quote 5K–M) and reaching for snacks which they know are a healthier option. In addition, participants referenced being more mindful of keeping active (Table 2, Quote, 5N), and although physical activity was not part of the trial, it was evident that some participants were conscious of the importance of exercise postpartum. Although this change in postpartum behavior was encouraging, participants did not explicitly state that this was an attempt to improve health outcomes such as prevention of type 2 diabetes.

Another driver behind the determined behavior to adhere to the diet was to avoid medication (Table 2, Quote 5O and P). This was a key motivator for a large proportion of women, and their self-awareness of eating habits and deliberate dedication to the diet ensured they could remain diet controlled. This was something that was evident even from the early stages after diagnosis, and some mentioned this as one of the main reasons to take part in the intervention and to truly engage in it. The participants who were able to stay diet controlled saw this as a true achievement, with some suggesting that this would not have been possible if left on their own to manage their diet (Table 2, Quote 5Q and R).

### Theme 6: research participation for awareness and impact

Many participants viewed the study as an opportunity to receive more support for dealing with GDM, but also reported that the opportunity to help others with GDM was a facilitator for them to participate in this study (Table 2, Quote 6A–C).

Participants recognized that being part of research is important to increase knowledge in GDM, and it was clear that a potential driver to taking part was to try to help other women in a similar situation. This was especially true for those who felt that more research on diabetes in pregnancy is required, and so they needed to play their part in advancing knowledge (Table 2, Quote 6D). Some also noted that this motivated them to adhere to study procedures, including the intervention and data collection, to provide useful data (Table 2, Quote 6E).

## Discussion

For participants in the DiGest trial, consuming a standard-energy or RE diet as part of whole-diet food provision during pregnancy was acceptable and associated with benefits to women’s emotional and physical health during a pregnancy affected by GDM. From our analysis, we developed 6 themes that were identified as key areas by taking part in the study highlighted. We observed that participants felt a significant amount of cognitive burden from a GDM diagnosis. Being in the study conferred benefits to their emotional and physical health, and the extra support and reassurance provided by the study research team were valuable. We also identified barriers such as hunger and facilitators such as convenience and health impacts on the mother and baby that served as motivators to participation, which can be used to further refine how implementing a dietary intervention would be more appealing to women with GDM. Importantly, we examined physical health/weight management and impact on pregnancy outcomes, and were able to identify participants’ perceptions of weight in pregnancy and postpartum, and whether they thought this was important for their future health. Participants had limited awareness of the potential benefits of an optimal diet and weight management in pregnancy on infant perinatal health and longer-term maternal cardiometabolic health, suggesting these areas should be prioritized for future work. The final theme of research participation highlighted the importance of raising awareness for GDM and how taking part in the research helped participants with their engagement in the intervention. All the themes identified here were present in both intervention arms, and no consistent patterns distinguished one group from the other. The findings are discussed here collectively rather than by study arm.

The first theme highlighted that participants often felt overwhelmed with the GDM diagnosis and had to cope with this during an already vulnerable and perhaps stressful period. It is well documented that women diagnosed with GDM often show signs of anxiety and stress at the time of diagnosis [13]. This was evident in the current cohort who described the shock of diagnosis and the emotional burden this had on them, which is important to consider when deciding on how best to support women during this time. This was emphasized by Feighan et al. [14], who suggested that it is just as important to have an insight into women’s emotional reaction to the condition as it is about being focused on the treatment aspects of GDM. Interestingly, through our interviews, we got the impression that while the diagnosis was an unpleasant surprise, it could be a trigger that helped women to reprioritize health and lifestyle, empowering them to take control of their pregnancy and health. Participating in the dietary intervention appears to further reinforce this shift; receiving nutritionally balanced foods tailored to their needs reduced the burden of dietary management and motivated women to remain engaged with the intervention.

Overall, it was evident that the participants enjoyed being in the study, and many aspects of the intervention were facilitators to participation and compliance. One of the main drivers facilitating adherence was convenience, because the meals were ready-to-eat with minimal preparation needed and no planning required for what would be appropriate for a GDM diet. This was a feature that had been identified in our previous qualitative study with healthcare professionals [9]. Indeed, others have shown that common barriers to healthy eating are often time and convenience, and this study suggested enablers are meal planning and organization [15].

This qualitative analysis has identified that participants often felt there was not enough support available for them after diagnosis, but that taking part in the study helped to address this. This is particularly important due to conflicting information available about the best ways to manage GDM. Therefore, the participants valued the extra monitoring and the diet planning provided by the study (“that little cheerleader behind the scenes”). However, after the pregnancy (and after the end of the study), many felt they went back to a state of lack of support at a time when they were even more overwhelmed with postpartum recovery and caring for a new baby. This highlights the need for further support right from diagnosis to at least early in the postpartum period. There is limited literature on optimizing postpartum care post-GDM. Previous work by Martinez and colleagues has highlighted barriers to postpartum care, such as demanding work schedules, limited childcare availability, and suboptimal transition to primary care [16]. This group suggests a 4-stage model to improve postpartum care, but this has yet to be implemented successfully.

Another facilitator was that many participants took part to help other women in the future. This stemmed from feelings of being unsupported, unclear on what the recommended dietary choices were, and being anxious after a GDM diagnosis. Participants were keen to make changes for the greater good, so that other women would have better experiences post-diagnosis in the future. Indeed, understanding the experiences of women will enable health care professionals to increase support and provide information on best practices to reduce the health implications of GDM [17].

We also identified several barriers to adherence, which were similar to what we previously found in the health care professional study [9]. Social aspects of food and disruption to the family meal times (eating differently from the rest of the family) were often cited as one of the main barriers to adherence to the study. This has been highlighted by Carolan and colleagues, who showed that having a restrictive diet for GDM management leads to certain social constraints that complicated their ability to self-manage their condition [18]. Despite this, many felt able to overcome this and feelings of hunger as they noted these dietary changes would only be required for a short period of time, with many indicating that the benefits outweighed the inconvenience of this temporary change. Future interventional studies should consider how to reduce these social constraints by offering options that could cater for the entire family or involving family members in the education about GDM diets.

The fifth theme covered the participants’ perceptions of their health impact on themselves and their baby as a motivator for intervention engagement. This was particularly focused on weight management and the impact this had on their baby. Many participants recognized that they were carrying extra weight during their pregnancy and, as such, on the whole were positive about weight loss, as long as their baby was maintaining a healthy growth trajectory. Current guidelines provided by the National Academy of Medicine suggest weight gain thresholds during pregnancy, but these are not refined to women with GDM [19]. Emerging evidence from the DiGest trial and others suggests that reduced gestational weight gain or weight loss in women with obesity or GDM can improve pregnancy outcomes [8,[20], [21], [22]]. Eleven of the 20 participants interviewed lost weight, which shows that it is an attainable target that women are prepared to achieve, provided they are given the support to do so.

For many women, avoiding medication such as metformin or insulin was a key benefit to women in the study. Most participants highlighted this as a key driver to maintain the diet for the duration of the study. It has previously been documented that women experience feelings of fear and guilt when they are unable to maintain optimal glucose using diet alone and require medication to control using their GDM [23,24]. Being able to reduce this fear through providing women with the diet empowered them to take control of their GDM and thus resulted in many of them being solely diet controlled, which some were unsure if they would have been able to do this under their own steam.

Improved glucose concentrations, consistent with milder hyperglycemia, boosted morale during the study, but many women were less clear on whether or not participation in the study improved infant outcomes. However, women did consistently consider that their baby’s outcomes were in line with healthy pregnancies, for example, by avoiding large-for-gestational age. Previous studies have shown that the main motivator for diet adherence in a GDM intervention study was their baby’s well-being [24], and although this was evident in the current study, the association of the diet playing a role in this was often not considered or reported. This is an important aspect to consider in future dietary interventions, although a strong focus on maintaining personal glucose control is key as a central motivator, there is a need to incorporate strategies to enhance participants’ understanding of how these changes can also affect fetal health.

Although women enjoyed taking part in the study, no participants mentioned the potential benefits of weight management on future health risks, such as progression to type 2 diabetes. National guidelines recommend that women with GDM receive information about future health risks, annual diabetes surveillance, and the diabetes prevention program. However, previous studies also highlighted the lack of awareness with the reasons behind this likely to be multifactorial including not receiving information from healthcare professionals about the risks, receiving it at an inappropriate time (when they are more focused on delivery of their baby) or in a non-ideal form (verbally which is easily forgotten), or lack of time, energy and finances to address this postnatally [10,23,25]. Therefore, this study emphasizes the need to raise awareness on the link between weight management during pregnancy (and postnatally) and future health risks associated with GDM, and to provide adequate and appropriate support to reduce these risks postnatally.

The findings in this study highlight several important key issues that have implications for the design of a scalable dietary intervention in pregnancy. Frequent contact with the research team provided reassurance and motivation; implementing such pathways within NHS-pathways without intensive research resources would be necessary to ensure this could be replicated at scale. In addition, meal provision and the study support were of great benefit in this intervention, but this ended postpartum, leading to a reversion of some participants to prepregnancy habits. Although providing meals for a longer period of time was not possible here, scaled dietary interventions could consider extending support mechanisms postpartum, such as through counseling or digital follow-ups, to reinforce sustained behavior change in a real-world setting. To translate these insights into broader implementation, future programs should integrate clear and accessible educational strategies, flexible and culturally appropriate meal options, and ongoing sustainable behavioral support postpartum to maximize adherence, participant satisfaction, and long-term maternal and fetal health outcomes.

### Strengths and limitations

We assessed the acceptability of an energy-restricted whole-diet intervention in pregnant women with GDM and a BMI ≥25 kg/m^2^. The cohort of participants interviewed for this evaluation study represented a good, diverse sample of the main study, with a range of education levels, parity, and distribution of weight loss compared with weight gain. The interviews were held online and, as such, reduced the barriers to inclusion for participants with competing family demands or busy work schedules. The interviews were held at various time frames from the study intervention, giving a broad representation of participants. The participants included for interview were from various NHS sites, highlighting that the support aspect identified as a key theme was not specific to 1 single hospital site. The interviewer was the study coordinator and a member of the study team, which meant that many of the participants had a rapport or were familiar with this member of the team. We have previously completed a similar study in health care professionals, and though there were some crossover in themes, this study provided an account of participants with lived experience of GDM and insight into the impact of receiving a GDM diagnosis, which has been invaluable. This qualitative study, along with the quantitative analysis performed as part of the main trial, suggests that there are clear benefits to being on the intervention, irrespective of trial arm.

There are several limitations identified in this study. Although we tried to reduce sampling bias, it is possible that the data captured during this qualitative process may only be of the most motivated women who were happy to share their experiences. Therefore, it is possible that the overwhelmingly positive feedback may not be representative of all participants. Furthermore, we cannot exclude the possibility of social desirability bias. The interviewer was also part of the study team who did many of the study visits, and so the participants may have been conscious about talking freely about their commitment to the study and the care they received. However, the variety of responses to the interview questions suggests that this was not the case and that the participants felt that they could give an honest review, even if this was negative. We included only 4 participants from a non-White background, which reduces the opportunity to assess the feasibility and acceptability of the trial in different cultural contexts. A majority of the interviews were done around a year into the postnatal period, but this was not for all participants; some of them were more than a year, which meant recall of experience was sometimes difficult as women had forgotten aspects of the intervention.

In conclusion, women found a meal replacement dietary intervention to be appealing and acceptable in a pregnancy with GDM, particularly where this is accompanied by higher levels of interpersonal support. Providing emotional support and reassurance during the time of GDM diagnosis and beyond is an important aspect to address, as women felt was limited outside of the intervention. This qualitative evaluation of the DiGest trial demonstrates that, despite barriers, provision of a whole-diet replacement was an acceptable tool to change dietary intake regardless of trial assignment, and has the potential to positively impact postnatal diet and lifestyle behaviors. Future research should explore ways to offer continued postpartum support for weight management and to reduce the risk of type 2 diabetes.

## Author contributions

The authors’ responsibilities were as follows – LCK, ALA, CLM: contributed to the conceptualization; LCK: contributed to the data curation; LCK, DLJ, SS, CLM: contributed to the methodology; LCK, SD: conducted formal analysis and wrote the original draft; LCK, SD, DLJ, SS, ALA, CLM: contributed to writing, reviewing and editing drafts; CLM: contributed to the funding acquisition, is the guarantor for this work, and takes responsibility for interpretation of the data; and all authors: read and approved the final manuscript.

## Data availability

Data described in the manuscript will not be made available due to the small number of participants, in order to protect participant anonymity.

## Funding

This research was funded by Diabetes United Kingdom (17/0005712; ISRCTN 65152174). CLM is also supported by the European Foundation for the Study of Diabetes -Novo Nordisk Foundation Future Leaders’ Award (NNF19SA058974). This work was supported by the NIH Research (NIHR), Cambridge Biomedical Research Centre (BRC), and the Leicester BRC. The views outlined here are those of the authors and not necessarily those of the NIHR or the Department of Health and Social Care**.** The funders had no role in the study design, data collection, analysis or interpretation of results. For the purpose of open access, the author has applied a Creative Commons Attribution (CC BY) license to any Author Accepted Manuscript version arising from this submission.

## Conflict of interest

The authors report no conflicts of interest.
