# Supplementary material for: Experience and Acceptability of a Reduced-Energy Whole-Diet Intervention in Women with Gestational Diabetes: a Qualitative Study of the Dietary Intervention in Gestational Diabetes Trial
Source: Curr Dev Nutr. 2026 Jan 5;10(2):107633. doi: 10.1016/j.cdnut.2025.107633 (PMC12860350; doi:10.1016/j.cdnut.2025.107633)
Supplement: Multimedia component 1 [file mmc1.docx]

**Supplementary Figures**

**Supplementary Figure 1:** Participant flow chart for the DiGest trial. Adapted from (8).


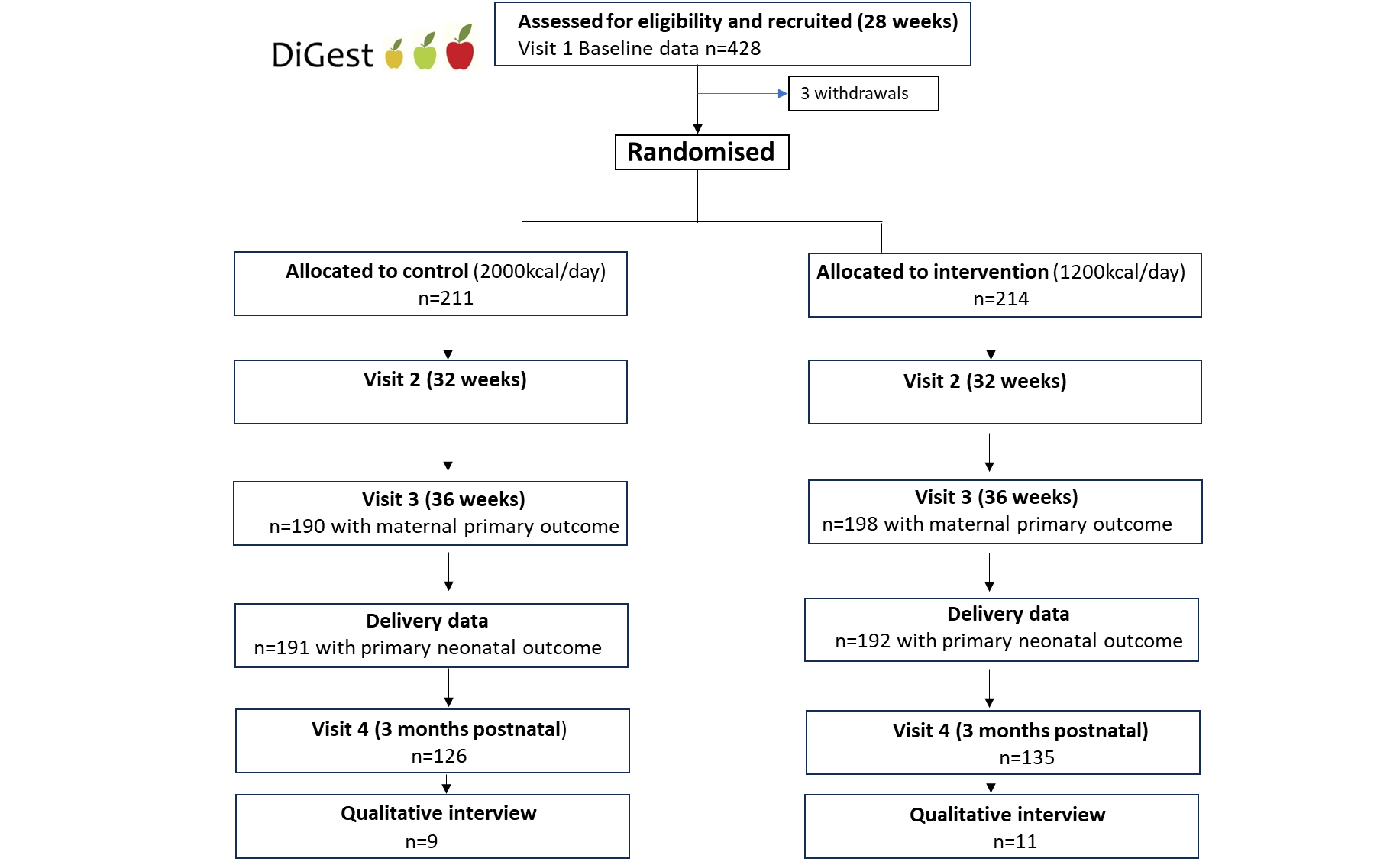


**Supplementary Figure 2:** Interview schedule for participants.

ACCEPTABILITY: intervention coherence

- What was your understanding of the study?
- What was the DiGest diet for?

ACCEPTABILITY: Affective attitude – in general

- Why did you decide to participate in this study? How did you weigh up the pros and cons of joining this study?

• How did you feel about being randomised to a standard or lower calorie dietbox?

- How did the experience match your expectations before you started the study?
- What other kinds of diets (slimming world, weight watchers, smartphone diet apps) have you used before?

ACCEPTABILITY: Affective attitude / ethicality – about weight changes / hunger

- Did you have any concerns about following the diet?
- Did you have concerns about weight loss or restricting weight gain in pregnancy?
- What happened to your weight while on the study? How did you feel about that?
- Do you feel taking part in the study has influenced weight after pregnancy?
- How did you feel about having a very structured routine of having the dietboxes alone? (Beneficial, miss cooking).
- Did you feel hungry during the study? How did you deal with that?
- Do you think the portion size was adequate- has it altered your perspective on portion control?

ACCEPTABILITY: Burden

- How easy was it to incorporate the dietbox meals into your everyday life, especially in terms of your
  - work routines
  - family meals
  - weekends
  - special occasions, birthdays or holidays
- How did you deal with times when you are eating with other people, such as at family events, at cafes or restaurants, or at your place of work?
- If any, which additional foods did you eat in addition to the dietbox? Why and when does this most commonly happen?
- What made it difficult to stick to dietbox foods?
- What would have made it easier for you to stick to the dietbox foods?
- How did your family, friends and colleagues react to you using the dietboxes? (
- What role did the healthcare team play in your use of the dietboxes? What did they do that helped or hindered you?
- Do you think you would be likely to continue with dietbox foods over a longer time frame (after your baby is born)?

ACCEPTABILITY: Ethical consequences

- Were there any side effects or problems with the intervention?

ACCEPTABILITY: Opportunity costs / Benefits e.g. upon finances

- What were the benefits of the study to you?
- What impact did taking part in the study have on your time and money? How did you feel about this?

ACCEPTABILITY: Perceived effectiveness & self-efficacy

- What impact did the study have on you and your baby?
- How easy did you find it to be part of the study and stick with the diet?
- How confident did you feel that you could keep going with the study diet?

ACCEPTABILITY: Overall summary

- What did you enjoy most about participating in the study?
- What did you enjoy least about participating in the study?

Would you recommend participating in the dietbox study to other pregnant women with gestational diabetes?

EVALUATION

- Regarding your dietbox what are your specific thoughts on
  - Ordering your weekly dietbox
  - The range of meal choices (too many, too few, prefer more fresh or frozen options?)
  - The dietbox delivery logistics
  - Cooking the dietbox meals
  - The smartphone app
  - The DiGest weighing scales

IMPLEMENTATION

- If we were to find that the dietboxes are beneficial for pregnancy in helping improve outcomes for women with gestational diabetes, how do you think we could implement this into NHS practice? (Prefer to have dietboxes, recipe cards, diet advice?)
- Do you think you could have followed the diet without the dietbox with dietary advice from the clinical team alone?
- How could we make the DiGest diet easier to adhere to/ more suitable for pregnant women with gestational diabetes?
- Do you have any other comments to make about your dietboxes or the study in general?

Thank you for sharing your experiences which will help us improve the dietbox experience for women with diabetes in pregnancy in the future.
